# α‐tACS over the somatosensory cortex enhances tactile spatial discrimination in healthy subjects with low alpha activity

**DOI:** 10.1002/brb3.2019

**Published:** 2021-01-06

**Authors:** Kei Saito, Naofumi Otsuru, Hirotake Yokota, Yasuto Inukai, Shota Miyaguchi, Sho Kojima, Hideaki Onishi

**Affiliations:** ^1^ Department of Physical Therapy Niigata University of Health and Welfare Niigata Japan; ^2^ Institute for Human Movement and Medical Sciences Niigata University of Health and Welfare Niigata Japan

**Keywords:** alpha rhythm, gamma rhythm, somatosensory cortex, spatial orientation, tactile perception, transcranial alternating current stimulation

## Abstract

**Introduction:**

Spontaneous oscillations in the somatosensory cortex, especially of the alpha (8 − 14 Hz) and gamma (60 − 80 Hz) frequencies, affect tactile perception; moreover, these oscillations can be selectively modulated by frequency‐matched transcranial alternating current stimulation (tACS) on the basis of ongoing oscillatory brain activity. To examine whether tACS can actually improve tactile perception via alpha and gamma modulation, we measured the effects of 10‐Hz and 70‐Hz tACS (α‐ and γ‐tACS) on the left somatosensory cortex on right‐finger tactile spatial orientation discrimination, and the associations between performance changes and individual alpha and gamma activities.

**Methods:**

Fifteen neurologically healthy subjects were recruited into this study. Electroencephalography (EEG) was performed before the first day, to assess the normal alpha‐ and gamma‐activity levels. A grating orientation discrimination task was performed before and during 10‐Hz and 70‐Hz tACS.

**Results:**

The 10‐Hz tACS protocol decreased the grating orientation discrimination threshold, primarily in subjects with low alpha event‐related synchronization (ERS). In contrast, the 70‐Hz tACS had no effect on the grating orientation discrimination threshold.

**Conclusions:**

This study showed that 10‐Hz tACS can improve tactile orientation discrimination in subjects with low alpha activity. Alpha‐frequency tACS may help identify the contributions of these oscillations to other neurophysiological and pathological processes.

## Graphical abstract

10‐Hz tACS can improve tactile orientation discrimination in subjects with low alpha activity. Conversely, 70‐Hz tACS had no effect on tactile orientation discrimination.

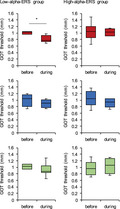

## 1 INTRODUCTION

Tactile sensation from peripheral somatosensory receptors is vital for fine dexterity by providing information on object texture, shape, weight, and orientation. Tactile information first arrives at the primary somatosensory cortex and is sequentially processed in the secondary somatosensory cortex and posterior parietal cortex (Inui et al., 2004). Efficient processing in the primary somatosensory cortex is critical for tactile discrimination (Li Hegner et al., 2015; Stilla et al., 2007; Van Boven et al., 2005; Zhang et al., 2005), and various noninvasive brain stimulation modalities targeting the somatosensory cortex have been shown to improve tactile discrimination performance. For instance, transcranial direct current stimulation (tDCS) of the primary somatosensory cortex can effectively reduce inhibitory circuit activity (Rehmann et al., 2016) and improve tactile grating orientation discrimination (Fujimoto et al., 2014, 2016; Ragert et al., 2008).

The cerebral cortex produces a complex oscillatory activity, which in turn is implicated in a variety of higher brain functions, such as sensory perception and memory. The alpha‐oscillation activity plays a special role in establishing the preparatory state for selective inhibition (Noonan et al., 2018 for review). In the visual cortex, increased alpha‐band power (event‐related synchronization, ERS) participates in the inhibitory control process (Rihs et al., 2007). Conversely, whether alpha ERS in the primary somatosensory cortex is involved in the inhibitory control process remains unclear. Haegens et al. (2011) demonstrated that an increased alpha‐oscillation power is significantly associated with a lower firing rate in monkey sensorimotor regions, suggesting that alpha ERS in the primary somatosensory cortex may play an important role in the inhibitory control processes for somatosensory information. Visual discrimination perception is associated with the alpha ERS induced by visual stimuli (Fründ et al., 2007). Based on these results, it would be conceivable that increased alpha ERS in the primary somatosensory cortex improves tactile grating orientation discrimination. However, increased gamma‐band power (gamma ERS) is involved in cortical activation. For example, Brookes et al. (2005) found that visual stimuli delivered using static checkboard induced both gamma ERS and sustained fields, reflecting successive excitation in the upper layers of the visual cortex. Ray et al. (2008) reported that the increased gamma‐band power induced by tactile input was associated with increased firing rate in the secondary somatosensory cortex. Collectively, gamma ERS in the primary somatosensory cortex participates in cortical activation in the primary somatosensory cortex. However, it is unclear whether gamma ERS in the primary somatosensory cortex is associated with tactile grating orientation discrimination. We found that the increased cortical activation in the primary somatosensory cortex induced by transcranial random noise stimulation was responsible for an improvement in the tactile grating orientation discrimination (Saito et al., 2019), suggesting that increased gamma ERS in the primary somatosensory cortex improves tactile grating orientation discrimination. Median nerve electrical stimulation was reported to induce ERS in a broad‐frequency band, including the alpha and gamma bands (Dockstader et al., 2008). Thus, we believe that median nerve electrical stimulation is optimal for inducing ERS in the alpha and gamma bands.

Transcranial alternating current stimulation (tACS) may be effective for increasing ongoing oscillatory brain activity (Zaehle et al., 2010; Neuling et al., 2012; 2013; Helfrich, Schneider, et al., 2014; Vossen et al., 2015; Kasten et al., 2016; Haberbosch et al., 2019). In addition, tACS at specific frequencies has been often demonstrated to alter various sensory functions (Kanai et al., 2008; Laczó et al., 2012; Helfrich, Knepper, et al., 2014; Kar & Krekelberg, 2014; Strüber et al., 2014; Müller et al., 2015; Riecke et al., 2015; Rufener et al., 2016; Rufener et al., 2016; Wöstmann et al., 2018; Baltus et al., ; Fusco et al., 2018). For example, Kanai et al. (2008) reported that tACS in the alpha‐frequency range (α‐tACS) over the visual cortex induced phosphenes in the dark in the absence of visual input. Furthermore, tACS at specific frequencies effectively altered somatosensory function (Feurra et al., 2011; Helfrich, Schneider, et al., 2014; Laczó et al., 2012; Neuling et al., 2012; Strüber et al., 2014). Feurra et al. (2011) reported that α‐tACS and tACS in the gamma‐frequency range (γ‐tACS) over the primary somatosensory cortex induced a tactile sensation in the absence of physical stimulation. Conversely, the effect of alpha oscillation in the somatosensory cortex on tactile discrimination remains unclear. Müller et al. (2015) reported that α‐tACS over the primary visual cortex effectively improved performance on a visual orientation discrimination task, suggesting that the increased alpha oscillations induced by α‐tACS over the primary visual cortex are associated with a better discrimination of orientation differences in visual stimulation. We predicted that the increased alpha oscillations induced by α‐tACS over the primary somatosensory cortex improve tactile grating orientation discrimination. Further, Neuling et al. (2013) reported that α‐tACS significantly increased the alpha activity in subjects with open eyes (low oscillation activity at the alpha frequency), whereas α‐tACS had no effect on the alpha activity in subjects with closed eyes (high oscillation activity at the alpha frequency), suggesting that α‐tACS increases alpha power in the primary somatosensory cortex in subjects with a low alpha power and improves tactile grating orientation discrimination. In contrast, γ‐tACS alters the perception and discrimination of various sensory stimuli (Baltus et al., 2018; Helfrich, Knepper, et al., 2014; Strüber et al., 2014). For example, Laczó et al. (2012) found that γ‐tACS over the primary visual cortex effectively improved visual contrast discrimination. Baltus et al. (2018) reported that tACS over the auditory cortex at an individual gamma frequency of +3 Hz enhanced temporal resolution in the auditory domain. Therefore, γ‐tACS may also increase gamma‐oscillation power, resulting in improved discrimination of sensory stimulation. Furthermore, the increased gamma‐oscillation power induced by γ‐tACS over the primary somatosensory cortex may improve tactile grating orientation discrimination. However, whether the effect of γ‐tACS on gamma oscillation is dependent on gamma‐oscillation power before the application of γ‐tACS remains unclear. As reported by Neuling et al. (2013), γ‐tACS may increase gamma activity in subjects with low gamma activity. This increase may also induce a greater gamma power in the primary somatosensory cortex in subjects with low gamma power and may improve tactile grating orientation discrimination.

The purpose of this study was to investigate the effects of α‐ and γ‐tACS on tactile orientation discrimination task performance, and the specific contributions of alpha and gamma oscillations. We hypothesized that α‐ and γ‐tACS over the primary somatosensory cortex improves the performance of tactile grating orientation discrimination and that changes in the tactile grating orientation discrimination performance induced by α‐tACS (reduced discrimination threshold) over the primary somatosensory cortex is associated with alpha ERS (lower alpha activity) before alpha‐tACS application. Finally, we hypothesized that the changes in tactile grating orientation discrimination performance induced by γ‐tACS (reduced discrimination threshold) over the primary somatosensory cortex are associated with gamma ERS (lower gamma activity) before γ‐tACS application.

## 2 EXPERIMENTAL PROCEDURES

### 2.1 Subjects

Fifteen neurologically normal male subjects were recruited into the study (age range, 20 − 23 years; mean ± standard deviation, 20.7 ± 0.8 years), of which 13 were right‐handed and two were left‐handed. The Edinburgh Handedness Inventory was used to determine the dominant hand.

### 2.2 Grating orientation discrimination task (GOT)

**FIGURE 1:**
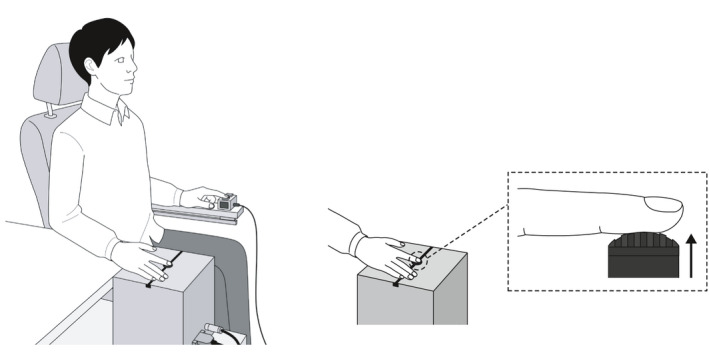
Tactile grating orientation discrimination task. Subjects were blindfolded and comfortably seated on a reclining chair. They received tactile stimulation at the tip of the right index finger from eight hemispherical domes with grooves of different width (3.0, 2.0, 1.5, 1.2, 1.0, 0.75, 0.5, and 0.35 mm) using a custom‐made device that automatically controls the up–down movements of the domes. The elevation speed of the hemispherical domes was set to 20 mm/s, and tactile stimulation duration was set to 1 s. The hemispherical domes were elevated for a further 1.5 mm after touching the tip of the right index finger. Subjects were first asked to judge the dome orientation relative to the long axis of the finger by pressing one button when they perceived the orthogonal orientation, and by pressing another button when they perceived the parallel orientation

Tactile spatial discrimination performance at the tip of the right index finger was assessed using a GOT, which is widely accepted as a robust measure of tactile spatial discrimination (Goldreich & Kanics, 2003; Ragert et al., 2008; Sathian et al., 1997). Tactile stimuli were delivered by eight hemispherical domes with different groove widths (3.0, 2.0, 1.5, 1.2, 1.0, 0.75, 0.5, and 0.35 mm) using a custom‐made device that automatically controls up–down dome movements (S‐16026; Takei Scientific Instruments Co. Ltd., Niigata, Japan) (Figure 1). The elevation speed of the hemispherical domes was set to 20 mm/s and tactile stimulation duration was set to 1 s based on a previous study (Saito et al., 2018, 2019). The hemispherical dome was elevated a further 1.5 mm after touching the tip of the right index finger. The subjects were then requested to judge the dome orientation relative to the long axis of the finger by pressing one button when they perceived the orthogonal direction and another when they perceived the parallel direction.

### 2.3 tACS

Transcranial ACS was delivered using a DC‐STIMULATOR PLUS instrument (Neuroconn, Germany) through a pair of saline‐soaked surface sponge electrodes (each, 5 × 5 cm). The tACS protocol was applied at 10 Hz (10‐Hz tACS) and 70 Hz (70‐Hz tACS) at a constant current intensity of 0.7 mA (peak‐to‐peak). One electrode was positioned 3 cm posterior to C3 of the international 10–10 system, and the other was positioned over the left shoulder. The stimulus waveform was sinusoidal without DC offset. The current was ramped up over the first 10 s of stimulation and then held constant for 15 min. For sham stimulation, one electrode was positioned 3 cm posterior to C3 of the international 10–10 system, and the other was positioned over the left shoulder. For sham stimulation, 10‐Hz tACS was ramped up over the first 10 s of stimulation and turned on for 30 s.

### 2.4 Experimental procedure

**FIGURE 2:**
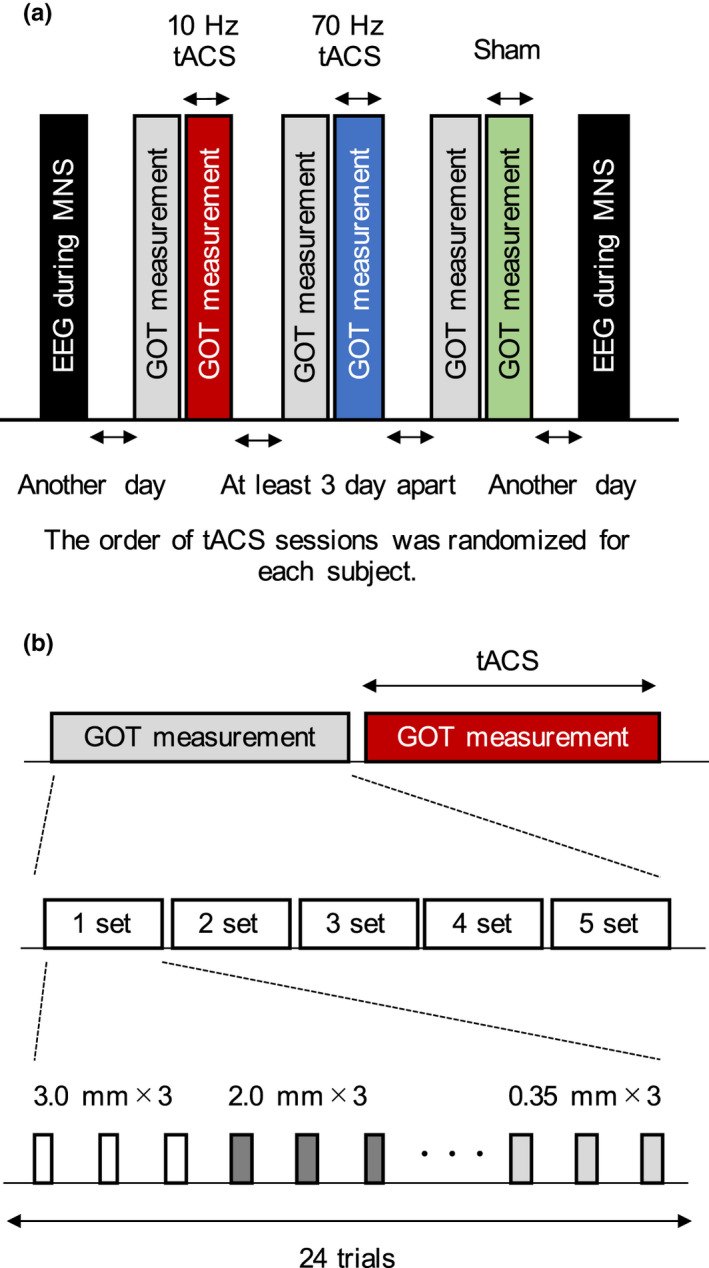
Scheme of the experiment.(A) EEG was measured during median nerve stimulation (MNS) before the first day and after the last transcranial alternating current stimulation (tACS) session. The tACS sessions were conducted (i) at 10 Hz (10‐Hz tACS), (ii) at 70 Hz (70‐Hz tACS), and (iii) under sham conditions. The order of tACS sessions was counterbalanced among the subjects. (B) GOTs were performed before and during the tACS sessions. The GOT consisted of five sets, each with 24 trials (3 trials for two orientations × 8 different groove widths). The domes with different groove widths were presented in the following order: 3.0, 2.0, 1.5, 1.2, 1.0, 0.75, 0.5, and 0.35 mm

Figure 2 shows the schema of the experiment. Subjects were comfortably seated on a reclining chair and blindfolded during the GOT analysis. All subjects received the following stimulus protocols: (a) 10‐Hz tACS, (b) 70‐Hz tACS, and (c) sham stimulation. The individual tACS sessions were separated by at least 3 days, and the protocol order was counterbalanced among the subjects. The EEG was recorded before the first day (mean ± standard deviation, 6.0 ± 4.0 days) and after the last tACS session (33.5 ± 35.8 days).

The GOT was performed before and during the application of tACS. The entire GOT comprised five sets of 24 trials (3 trials × 8 different groove widths). The groove widths of the dome were presented in the order of 3.0, 2.0, 1.5, 1.2, 1.0, 0.75, 0.5, and 0.35 mm, while the orientations were presented randomly. Over the five sets, each dome was presented 15 times (seven times in the orthogonal direction and eight times in the parallel direction, or vice versa).

### 2.5 Electroencephalography (EEG) recording and analysis

EEG was recorded for 5 min during electrical stimulation of the right median nerve of the wrist. Electrical stimulation was continuously delivered for 5 min using the following stimulation parameters: 0.3 Hz, pulse width of 0.2 ms, and stimulus intensity 1.2 times the motor threshold. The motor threshold was defined as the lowest intensity that induced a visible twitch in the thenar muscle. The EEG signals were sampled at 2,500 Hz from 31 Ag–AgCl electrodes (Brain Vision, Brain Products, Germany) positioned at Fp1, Fp2, F3, F4, FC1, FC2, FC5, FC6, FT9, FT10, Cz, C3, C4, T7, T8, CP1, CP2, CP5, CP6, Pz, P3, P4, P7, P8, TP9, TP10, Oz, O1, and O2 according to the international 10–10 system. A reference electrode was placed at Fz. The EOG was recorded from Ag–AgCl electrodes affixed above and below the right eye, at 1 cm lateral from the outer canthus. The impedance at each electrode was kept below 5 kΩ.

Multiple EEG parameters were analyzed using EEGLAB 14.1.2b (https://sccn.ucsd.edu/eeglab/index.php) (Delorme and Makeig 2004) programmed in MATLAB (version 2017a; MathWorks, Natick, MA, USA). Briefly, EEG records were downsampled to 500 Hz, followed by off‐line band‐pass filtering at 1 − 100 Hz using finite impulse response filtering. Line noise (50 Hz) was attenuated using the Cleanline EEGLAB plugin. An extended infomax independent component analysis with a natural gradient was performed using EEGLAB, to obtain 32 independent components (ICs) from each subject, followed by the removal of ICs for blinking, eye movement, and electrocardiogram signals. In addition, we visually checked EEG data that might have contained muscle activity and removed these data. The EEG data recorded during stimulation were segmented into epochs from 1,000 ms prior to 2000 ms after the peripheral electrical stimulus onset. The epochal EEG data for each electrode were corrected using their respective baseline averages (baseline from −1,000 to 0 ms).

A time–frequency analysis was performed on the EEG epochs induced by electrical stimulation using the Morlet wavelets to calculate event‐related spectral perturbations at the C3 electrode. For analysis of alpha ERS, 41 linearly spaced frequencies from 5 to 30 Hz were calculated every 2 ms from 1,000 ms before to 2000 ms after the onset of the stimulation, with a wavelet cycle of 2 at 5 Hz and 6 at 30 Hz. For the analysis of gamma ERS, 61 linearly spaced frequencies from 25 to 100 Hz were calculated every 4 ms from 1,000 ms before to 2000 ms after the onset of the stimulation, with a wavelet cycle of 5 at 25 Hz and 10 at 100 Hz. The data at each frequency resolution (alpha band, 0.62 Hz; gamma band, 2.5 Hz) obtained from the time–frequency analysis were corrected using their respective baseline averages (baseline from −700 to −200 ms). The data from 7.5 to 13.5 Hz corrected to the individual baseline average were averaged to obtain the alpha ERS, while the data from 60 to 80 Hz corrected to the individual baseline average were averaged to obtain the gamma ERS. The positive peak amplitude was calculated between 1 and 349 ms after the stimulation, to obtain event‐related synchronization in the alpha band (alpha ERS), and between 1 and 90 ms after the stimulation, to obtain the gamma ERS. The time point at which the positive peak amplitude was calculated was determined based on the individual latency of ERS. In this study, the peak latency of alpha ERS was 119.9 ± 109.4 ms, and the peak latency of gamma ERS was 10.7 ± 8.6 ms. Thus, we obtained alpha ERS between 1 and 349 ms and gamma ERS between 1 and 90 ms in our study.

### 2.6 Behavioral data analysis

The proportion of correct responses at each groove width (% of total trials) was determined, and the grating orientation discrimination threshold was derived for each subject using these results. Briefly, groove width was plotted against the percentage of correct responses and fitted by logistic regression based on a generalized linear model. The linear regression coefficient was calculated using the following equation.

$$K_{1}+K_{2}X=\log\;\left[Y/\left(1\;‐\;Y\right)\right],$$

where K is the linear regression coefficient, X is the grating width, and Y is the correct response rate.

The grating orientation discrimination threshold was then calculated using the following equation.

$$\text{Threshold}=\left\{\text{log [}0.75/\left(1\;‐\;0.75\right)]‐K_{1}\right\}/K_{2},$$

where K_1_ and K_2_ are linear regression coefficients.

We also examine the correlations between EEG parameters and the changes in grating orientation discrimination induced by tACS. The change in the grating orientation discrimination threshold induced by tACS was calculated using the following equation.

$$\Delta \text{GOT discrimination threshold}=\left({\text{Threshold}}_{\text{during}}‐{\text{Threshold}}_{\text{before}}\right)/{\text{Threshold}}_{\text{before}}\times 100,$$

where Threshold_during_ is the grating orientation discrimination threshold during tACS and Threshold_before_ is the grating orientation discrimination threshold before tACS.

Moreover, the 15 subjects were divided into two groups according to the alpha ERS measured before the first tACS session: a low‐alpha‐ERS group, with an alpha ERS smaller than the median value, and a high‐alpha‐ERS group, with an alpha ERS greater than the median value.

### 2.7 Questionnaires

The subjective experiences of the participants during tACS and sham stimulation were evaluated by a questionnaire using a numeric rating scale (NRS) 1 min after tACS initiation and sham stimulation, to rate their levels of itching, tingling, and phosphenes. Subjects used an 11‐point scale, with 0 representing the absence of sensation and 10 being the strongest sensation.

### 2.8 Statistical analysis

We first examined whether the group data for alpha and gamma ERS, grating orientation discrimination threshold, Δ grating orientation discrimination threshold, and grating orientation discrimination threshold in the low‐alpha‐ERS and high‐alpha‐ERS groups fit normal distributions using the Shapiro–Wilk test. To examine the day‐to‐day variation in EEG parameters, we compared individual alpha and gamma ERS values before and after tACS sessions using a paired *t* test only if the two data to be compared were both parametric; otherwise, we used the Wilcoxon signed‐rank test. The grating orientation discrimination threshold was analyzed by two‐way repeated analysis of variance (ANOVA) using stimulus condition (10‐Hz tACS, 70‐Hz tACS, or sham stimulation) and time (before or during stimulation) as the main factors. Furthermore, we analyzed the correlations between the EEG parameters (alpha and gamma ERS) measured before the first tACS session and the Δ grating orientation discrimination threshold using Pearson's correlation coefficient only if the two data to be tested for correlation were both parametric; otherwise, we used Spearman's rank correlation coefficient. Finally, the 15 subjects were divided into two group according to the alpha ERS value before tACS sessions: the low‐alpha‐ERS and high‐alpha‐ERS groups. We compared the grating orientation discrimination threshold before tACS sessions in the low‐alpha‐ERS group and the high‐alpha‐ERS group using the Wilcoxon signed‐rank test. In addition, we compared the grating orientation discrimination threshold before and during tACS or sham stimulation in the low‐alpha‐ERS and high‐alpha‐ERS groups using the Wilcoxon signed‐rank test depending on the results of the Shapiro–Wilk test.

All statistical analyses were performed using SPSS ver25 for Mac.

Statistical significance was set at *p* < .05. Corrected *p* values were always reported. All corrected *p* values > 1 were reported as *p* = 1.

### 2.9 Ethics Statement

The studies were performed in accordance with the Declaration of Helsinki and were approved by the ethics committee of Niigata University of Health and Welfare.

## 3 RESULTS

### 3.1 Day‐to‐day variation in EEG parameters

**FIGURE 3:**
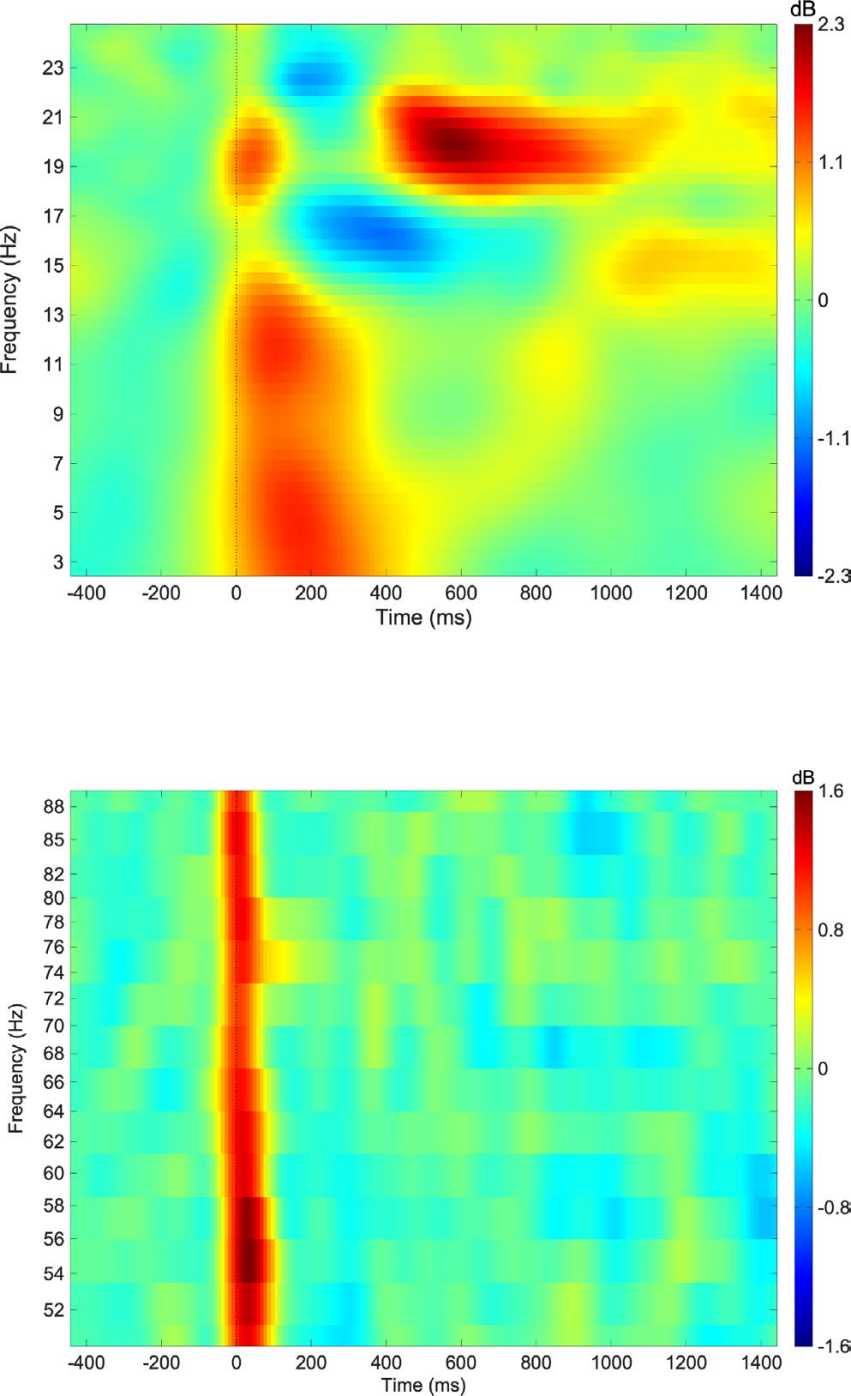
Electroencephalography (EEG) parameters used to assess alpha and gamma activities in the somatosensory cortex. The average time–frequency map of ERSPs in the frequency band from 1 to 30 Hz before tACS sessions (upper panel) and in the frequency band from 50 to 90 Hz (lower panel)

There were no significant differences in alpha (8 − 14 Hz) ERS and gamma (60 − 80 Hz) ERS after the final tACS session compared with the values recorded before the first tACS session (all *p* > .05, paired *t* test or Wilcoxon's signed‐rank test). Figure 3 presents the average alpha and gamma event‐related perturbation (ERP) waveform of channel C3 during median nerve stimulation.

### 3.2 Effect of tACS on perceptual performance in the GOT

**FIGURE 4:**
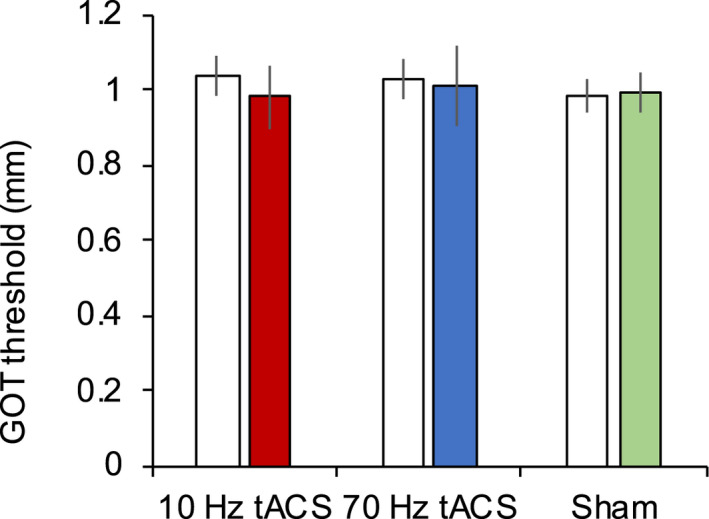
Effects of tACS on the tactile discrimination threshold (mean ± standard error). Transcranial alternating current stimulation had no effect on the grating orientation discrimination threshold. White boxes, before tACS; colored boxes, during tACS

**FIGURE 5:**
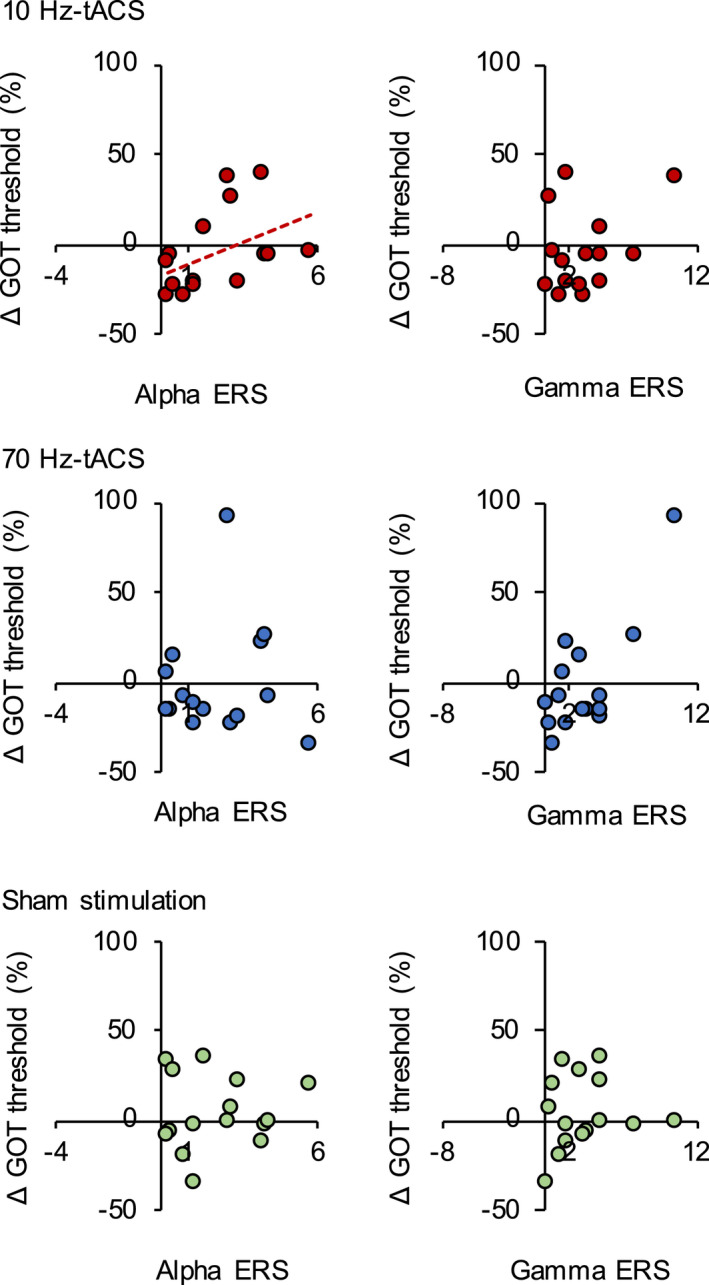
Improved tactile discrimination threshold by 10‐Hz tACS in individuals with lower alpha event‐related synchronization. Correlation between each EEG parameter and the changes in the grating orientation discrimination threshold induced by 10‐Hz tACS (upper) for each subject (*n* = 15); between each EEG parameter and the changes in the grating orientation discrimination threshold induced by 70‐Hz tACS (middle) for each subject (*n* = 15); and between each EEG parameter and the changes in the grating orientation discrimination threshold induced by sham stimulation (lower) for each subject (*n* = 15). A positive correlation was observed between α‐ERS and the changes in the grating orientation discrimination threshold induced by 10‐Hz tACS

Two‐way repeated measures ANOVA indicated no significant effect of stimulus condition (10‐Hz tACS, 70‐Hz tACS, or sham stimulation) (*F*_(1.384, 19.376)_ = 0.201, *p* = .737; Greenhouse − Geisser corrections for degrees of freedom) or time (*F*_(1, 14)_ = 0.185, *p* = .673) on grating orientation discrimination threshold as well as no significant interaction between stimulus condition and time (*F*_(2, 28)_ = 0.264, *p* = .770) (Figure 4). The associations between EEG parameters and the change in grating orientation discrimination threshold during tACS are summarized in Figure 5 and Table 1. There was a positive correlation between alpha ERS and the change in discrimination threshold induced by 10‐Hz tACS (Spearman's *R* = 0.579, *p* = .024). There was no significant correlation between the grating orientation discrimination threshold change induced by 10‐Hz tACS and gamma ERS (*p* > .05). Moreover, there was no significant correlation between the grating orientation discrimination threshold change induced by 70‐Hz tACS and alpha and gamma ERS (all *p* > .05). Similarly, there were no significant correlations between the change in the grating orientation discrimination threshold induced by sham stimulation and any of the EEG parameters (all *p* > .05).

**TABLE 1:** Pearson's product–moment correlation and Spearman's rank correlation coefficient (*R*) between EEG parameters and the changes in the grating orientation discrimination threshold induced by tACS

	EEG parameters	Correlation coefficient (*r*)	*p*‐value
10‐Hz tACS	Alpha ERS	.579	.024*
	Gamma ERS	.193	.491
70‐Hz tACS	Alpha ERS	−.025	.93
	Gamma ERS	.432	.108
Sham stimulation	Alpha ERS	.069	.806
	Gamma ERS	.236	.398

* *p* < .05.

**FIGURE 6:**
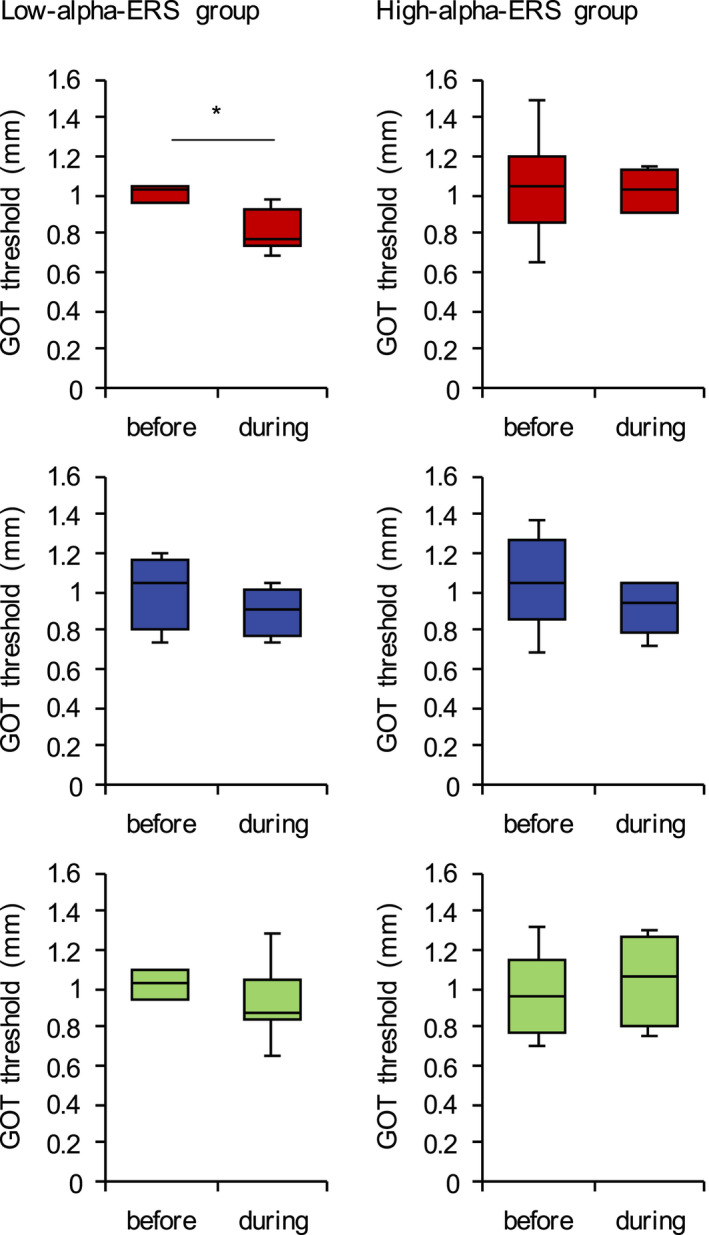
Effects of tACS on the tactile discrimination threshold in each group. Effect of 10‐Hz tACS (upper), 70‐Hz tACS (middle), and sham stimulation (lower) on the tactile discrimination threshold in each group. In the low‐alpha‐ERS group, the grating orientation discrimination threshold during 10‐Hz tACS was significantly lower than the baseline threshold

Seven subjects were included in the low‐alpha‐ERS group, and the remaining eight subjects were included in the high‐alpha‐ERS group. We found no significant difference in the tactile discrimination threshold before tACS application between the low‐ and high‐alpha‐ERS groups (Table 2). In the low‐alpha‐ERS group, the grating orientation discrimination threshold during 10‐Hz tACS was significantly lower than the baseline threshold (*U* = −2.747, *p* = .004 (uncorrected), *p* = .024 (corrected); Mann–Whitney *U* test) (Figure 6). We found no significant effects of 10‐Hz tACS on the grating orientation discrimination threshold in the high‐alpha‐ERS group (*U* = −0.105, *p* = .916 (uncorrected), *p* = 1.000 (corrected); Mann–Whitney *U* test). Furthermore, we found no significant effects of 70‐Hz tACS on the grating orientation discrimination threshold in the high‐ and low‐alpha‐ERS groups (high‐alpha‐ERS group: *U* = −0.735, *p* = .505 (uncorrected), *p* = 1.000 (corrected), Mann–Whitney *U* test; low‐alpha‐ERS group: *U* = −1.214, *p* = .259 (uncorrected), *p* = 1.000 (corrected), Mann–Whitney *U* test). Similarly, we found no significant effects of sham stimulation on the grating orientation discrimination threshold in both the high‐ and low‐alpha‐ERS groups (high‐alpha‐ERS group: *U* = 0.525, *p* = .645 (uncorrected), *p* = 1.000 (corrected), Mann–Whitney *U* test; low‐alpha‐ERS group: *U* = −1.086, *p* = .318 (uncorrected), *p* = 1.000 (corrected), Mann–Whitney *U* test).

**TABLE 2:** Tactile discrimination threshold before tACS application in the low‐ and high‐alpha‐ERS groups

	Low‐alpha‐ERS group	High‐alpha‐ERS group	Statistic	*p*‐value
10‐Hz tACS	1.02 (0.98–1.04)	0.99 (0.90–1.14)	0.231	.867
70‐Hz tACS	1.05 (0.86–1.15)	0.94 (0.88–1.18)	0.579	.613
Sham stimulation	1.03 (0.99–1.08)	0.93 (0.82–1.13)	−0.405	.694

### 3.3 Subjective sensations during tACS

All subjects tolerated the applied current under each stimulation condition, and there were no interruptions caused by adverse effects. Table 3 summarizes the subjective ratings for itching, tingling, and phosphenes. The median NRS values were all zero, suggesting that these sensations were absent or very mild.

**TABLE 3:** Sensations induced by tACS and sham stimulation

Sensation	10‐Hz tACS	70‐Hz tACS	Sham stimulation
Itching	0 (0–0)	0 (0–0)	0 (0–0)
Tingling	0 (0–0)	0 (0–0.5)	0 (0–0)
Phosphenes	0 (0–0)	0 (0–0)	0 (0–0)

Note

Values are the median (interquartile range).

## 4 DISCUSSION

This study demonstrated that alpha‐frequency tACS of the somatosensory cortex can enhance tactile spatial perception and that the magnitude of the effect is dependent on the individual alpha ERS measured before the tACS sessions. The 10‐Hz tACS protocol decreased the grating orientation discrimination threshold primarily in subjects with low‐alpha‐ERS. Conversely, 70‐Hz tACS had no effect on the grating orientation discrimination threshold. In summary, 10‐Hz tACS (α‐tACS) can improve tactile discrimination, especially in subjects with low alpha activity.

### 4.1 Effect of 10‐Hz tACS on tactile discrimination

In the present study, we hypothesized that α‐tACS over the primary somatosensory cortex would improve the performance of tactile grating orientation discrimination. However, we found that 10‐Hz tACS had no significant effect on tactile grating orientation discrimination. Previous studies have reported similar findings (Otsuru et al., 2019; Wittenberg et al., 2019). This discrepancy may be explained by an obscured overall effect of tACS caused by subject heterogeneity. Several previous studies have shown that alpha‐oscillation power is typically increased during α‐tACS (Helfrich, Schneider, et al., 2014), as well as after tACS (Haberbosch et al., 2019; Kasten et al., 2016; Neuling et al., 2012, 2013; Vossen et al., 2015; Zaehle et al., 2010). Conversely, a recent study has demonstrated that α‐tACS decreases alpha‐oscillation power (Gundlach et al., 2017). α‐tACS may increase alpha‐oscillation power in one subject, while decreasing it in another subject. In addition, visual discrimination task performance is associated with alpha‐oscillation power in the visual cortex (Fründ et al., 2007; Samaha et al., 2017). Therefore, it is plausible that the overall effect of 10‐Hz tACS over the primary somatosensory cortex on alpha‐oscillation power is masked by subject heterogeneity, thus obscuring its overall effect on tactile discrimination perception. Here, 10‐Hz tACS improved tactile discrimination in subjects with low alpha ERS, while 10‐Hz tACS had no effect on tactile discrimination perception in subjects with high alpha ERS. The correlation between the effect of α‐tACS on tactile discrimination perception and alpha‐oscillation activity remains to be investigated. Neuling et al. (2013) found that α‐tACS significantly increased alpha activity in subjects with open eyes (low oscillation brain activity at alpha frequencies), but had no effect on alpha activity in patients with closed eyes (high oscillation brain activity at alpha frequencies). Collectively, 10‐Hz tACS may selectively increase alpha ERS in subjects with low alpha ERS, thereby strengthening the inhibition of tactile information irrelevant to tactile discrimination. This improvement may be related to the suppression of inhibitory activity in the primary sensory cortex, as Haegens et al. (2011) reported that the firing frequency of cortical pyramidal cells was reduced by increased alpha oscillations, and numerous reports have demonstrated that tACS can enhance cortical rhythms, including alpha oscillations. Lakshminarayanan et al. (2015) found that the detection of target tactile information was impaired by delivering tactile information irrelevant to the task, suggesting that the inhibition of irrelevant tactile inputs may improve tactile discrimination perception. It is plausible, therefore, to contend that 10‐Hz tACS over the primary somatosensory cortex may act to decrease tactile input irrelevant to the GOT. Rihs et al. (2007) reported that alpha ERS represented inhibition of somatosensory information irrelevant to a visual detection task, in accordance with this notion.

Alternatively, we found no correlation between the tactile discrimination induced by 10‐Hz tACS and gamma ERS. This may be explained by the involvement of different mechanisms in alpha‐ and gamma‐oscillation activity. Rihs et al. (2007) found that increased alpha ERS is involved in the inhibitory control process, whereas several studies have shown that increased gamma ERS is involved in cortical activation (Brookes et al., 2005; Ray et al., 2008). Furthermore, α‐tACS has been shown to increase brain oscillations in a stimulus‐frequency‐specific manner. Zaehle et al. (2010) reported that tACS at the participant's individual alpha frequency (iAF) significantly increased the individual alpha power, but did not change the ongoing oscillatory brain activity in the frequency band of iAF − 5Hz to iAF − 3Hz and iAF + 3 Hz and iAF + 5 Hz. These results indicated that α‐tACS may be strongly related to ongoing oscillatory brain activity in the alpha band, but not the gamma band.

### 4.2 Effects of 70‐Hz tACS on tactile discrimination

The 70‐Hz tACS protocol had no effect on tactile discrimination. This differential effect may be explained by effects of 70‐Hz tACS on gamma activity. While numerous studies have reported that tACS increases ongoing oscillatory brain activity within the stimulation frequency band (Zaehle et al., 2010; Neuling et al., 2012; 2013; Helfrich, Schneider, et al., 2014; Vossen et al., 2015; Kasten et al., 2016; Haberbosch et al., 2019), Helfrich, Knepper, et al. (2014) found that γ‐tACS had no effects on gamma activity. Collectively, α‐tACS effectively increases the ongoing oscillatory brain activity in the alpha frequency, whereas γ‐tACS may have no effect on ongoing oscillatory brain activity in the gamma frequency. Furthermore, Helfrich, Knepper, et al. (2014) found that γ‐tACS significantly decreased alpha activity, suggesting that 70‐Hz tACS actually weakens the inhibition of tactile information irrelevant to tactile discrimination by suppressing alpha activity. In turn, this suppression of alpha activity induced by 70‐Hz tACS may cancel the effect of increased gamma activity on tactile discrimination perception, even if 70‐Hz tACS increases the gamma activity. Therefore, it is plausible to suggest that 70‐Hz tACS over the primary somatosensory cortex does not affect tactile discrimination.

### 4.3 Limitations

This study had several limitations. We performed EEG and GOT measurements on different days, to minimize the impact of long experimental procedures, because if EEG and GOT were measured on the same day, the experiment time would be long and the attention required for GOT measurements would be affected. Thus, we cannot determine the correlation between the effect of tACS on tactile discrimination perception and EEG data just before performing GOTs. In addition, the cortical oscillation activity in the alpha‐ and gamma‐band frequencies induced by tACS may contribute to changes in tactile discrimination perception. Thus, future studies examining cortical oscillation activity during tACS application employing EEG or magnetoencephalography are warranted for clarifying whether the changes in cortical oscillation activity induced by tACS reflect changes in tactile discrimination perception. Moreover, the number of subjects recruited into the present study was not large and the subjects were divided into two groups. In addition, the study participants included two left‐handed individuals, both of whom were in the low‐alpha group. Considering that discrimination thresholds are usually higher for the nondominant hand than they are for the dominant hand, measuring the discrimination threshold of the nondominant hand may be responsible for the lowered discrimination threshold recorded during tACS. Future studies examining the effects of tACS on tactile perception in a larger cohort of right‐handed subjects are warranted. Last, we measured EEG during electrical stimulation of the median nerve to induce alpha and gamma ERS. Electrical artifacts generated by median nerve stimulation may affect the ERS value. Studies employing multiple methodologies (e.g., a paired‐pulse protocol using paired‐pulse stimuli with an interpulse interval to examine the inhibitory mechanism in the primary somatosensory cortex, or measurement of power spectral density with fast Fourier transform on EEG recorded at rest) are planned to assess the correlation between the effect of tACS on tactile discrimination perception and cortical activity.

## 5 CONCLUSION

The application of 10‐Hz tACS over the primary somatosensory cortex reduced the discrimination threshold in individuals with lower alpha activity. Therefore, 10‐Hz tACS can improve tactile discrimination depending on the ongoing oscillatory brain activity in healthy subjects.

## CONFLICT OF INTEREST

The authors declare that the research was conducted in the absence of any commercial or financial relationships that could be construed as a potential conflict of interest.

## AUTHOR CONTRIBUTIONS

KS and HO conceived the study and designed the experiments. KS and HY carried out the experiments. NO interpreted the data. YI, SK, and SM helped write the manuscript.

### Peer Review

The peer review history for this article is available at https://publons.com/publon/10.1002/brb3.2019.

### DATA AVAILABILITY STATEMENT

The data used to support the findings of this study is available from the corresponding author upon request.
